# RNA Interference for Improving Disease Resistance in Plants and Its Relevance in This Clustered Regularly Interspaced Short Palindromic Repeats-Dominated Era in Terms of dsRNA-Based Biopesticides

**DOI:** 10.3389/fpls.2022.885128

**Published:** 2022-05-13

**Authors:** Koushik Halder, Abira Chaudhuri, Malik Z. Abdin, Manoj Majee, Asis Datta

**Affiliations:** ^1^National Institute of Plant Genome Research, New Delhi, India; ^2^Centre for Transgenic Plant Development, Department of Biotechnology, School of Chemical and Life Sciences, Jamia Hamdard University, New Delhi, India

**Keywords:** biopesticides, biotic stress, CRISPR/Cas9, dsRNAs, gene edited, GMO, RNAi

## Abstract

RNA interference (RNAi) has been exploited by scientists worldwide to make a significant contribution in the arena of sustainable agriculture and integrated pest management. These strategies are of an imperative need to guarantee food security for the teeming millions globally. The already established deleterious effects of chemical pesticides on human and livestock health have led researchers to exploit RNAi as a potential agri-biotechnology tool to solve the burning issue of agricultural wastage caused by pests and pathogens. On the other hand, CRISPR/Cas9, the latest genome-editing tool, also has a notable potential in this domain of biotic stress resistance, and a constant endeavor by various laboratories is in progress for making pathogen-resistant plants using this technique. Considerable outcry regarding the ill effects of genetically modified (GM) crops on the environment paved the way for the research of RNAi-induced double-stranded RNAs (dsRNA) and their application to biotic stresses. Here, we mainly focus on the application of RNAi technology to improve disease resistance in plants and its relevance in today’s CRISPR-dominated world in terms of exogenous application of dsRNAs. We also focused on the ongoing research, public awareness, and subsequent commercialization of dsRNA-based biocontrol products.

## Introduction

Agricultural sector is one of the major contributors to the economy of lower-middle-income countries such as India. Yet each year, biotic stresses such as insects, fungi, bacteria, and nematodes cause substantial losses in crop productivity and overall agricultural production. Conventional breeding methods proved to be time-consuming and inefficient to come up with disease-resistant smart crops for a sustainable agricultural system, calling for the need of plant biotechnological interventions. RNA interference (RNAi), discovered more than a decade ago, is one of the central players of gene regulation and mounting defense against viral infections in plants. Through RNAi pathway, long double-stranded RNAs (dsRNA) are processed into small interfering RNAs (siRNA), which specifically binds and cleaves the targeted viral messenger RNAs (mRNA) in the cytosol, leading to an effective protection of plants (Mezzetti et al., 2020). This potential multipurpose tool is being exploited on a large scale to interfere with the pest–pathogen genes by processes such as host-induced gene silencing (HIGS) (Nowara et al., 2010) and spray-induced gene silencing (SIGS) (Koch et al., 2016) and can make a remarkable contribution in the domain of integrated pest management (Koch et al., 2019). Since the conventional RNAi method involves the production of genetically modified (GM) crops where exogenous dsRNAs are expressed to induce RNAi pathway within, anti-GMO groups has raised concerns regarding the safe consumption of these plant products carrying heterologous genetic material mostly from bacteria, fungi, etc. (Zotti et al., 2018). Although gradually, the stigma against RNAi-based pest–pathogen-resistant plants is reducing worldwide as quite a lot of virus-resistant plants (Squash, Papaya, Plum) have gained approval to be cultivated outside the European Union (for example, in North and South American countries, *cis*/transgenic crops which do not carry antibiotic resistance genes are not considered as GMOs; Waltz, 2015; Khalid et al., 2017; Limera et al., 2017). On the other hand, clustered regularly interspaced short palindromic repeats (CRISPR/Cas), the most efficient genome editing tool, is in the headlines now as required insertion and deletion (in/del) of nucleotides can be done in the genomic loci very precisely and it can be applied to generate stable knockout plant lines with improved disease resistance against pests and pathogens (Charpentier and Dounda, 2013; Mat Jalaluddin et al., 2019). In many countries, CRISPR/Cas-food crops are not categorized under GMO status because even if the whole procedure involves a transient transgenic state, eventual sexual propagation leads to transgene-free crops by the law of segregation (Schenke and Cai, 2020). Although on a separate note, in case of woody plants, transgene can remain unmodified for over 20 years (Walter et al., 2005) and sexual propagation is not an option for obtaining homozygous single-copy clone. Vegetative propagation on a large scale for field plantation followed by copy number determination using inverse polymerase chain reaction (PCR) can be a suitable solution for ensuring genetic stability of transgenes in woody plants (Stefano et al., 2016). Plant biotechnology field has upheld many instances showing that RNAi is unique in many respect catering to a broader domain of requirements of breeders for improved crop variety as compared to CRISPR/Cas (Mezzetti et al., 2020). For instance, the most recent and talked about innovation in the field of pest management is the exogenous (topical) application of dsRNAs as the most effective strategy for gene silencing (San Miguel and Scott, 2016; Worrall et al., 2019). It is being considered worldwide as a more practical and realistic method as biopesticides since a lot of noteworthy researches have been done to improve several aspects of exogenously applied dsRNAs such as stability, efficacy, and persistence. The modes of application are foliar sprays, seed treatments, root drenching, and many more; all are commercially lucrative options because of the comparatively low production cost, high specificity and the most important aspect is improved biosafety than other categories of biopesticides and chemical pesticides (Zotti et al., 2018; Cagliari et al., 2019; Bramlett et al., 2020; Rodrigues and Petrick, 2020).

In this review, we will delve deep into the details of both RNAi and CRISPR/Cas technology, how these technologies are being used for improving disease resistance in plants against pests such as insects, fungi, and nematodes and how RNAi technology is still relevant in terms of exogenous application of dsRNAs in this new era of CRISPR/Cas.

## Two Ground-Breaking Gene-Editing Tools

### RNA Interference: The Revolutionary Gene-Silencing Technique

Target-specific gene silencing has gained immense popularity among the scientific community over the years. Out of many gene-silencing techniques, RNAi has been proven to be very effective in targeting and degrading a particular mRNA of a gene. When an organism is exposed to a foreign genetic material such as DNA or RNA, it immediately started mounting an immune response against it. At the very core of such type of sequence directed immune response is RNAi. In the early 1995, Guo and Kemphues tried to use the antisense-strand targeting *Caenorhabditis elegans par-1* gene to silence its expression, which is also known as antisense-mediated gene silencing (Guo and Kemphues, 1995). They found not only the exogenous antisense strand but also the sense RNA strand was able to induce silencing. The observation by Fire et al. (1998) first led to the idea that dsRNA is a potent trigger for RNAi-mediated gene silencing, and it is much more effective as compared to a single-stranded RNA. They coined the term “RNA-interference” because they showed for the first time that exogenous introduction of RNA (both sense and antisense) can interfere with the function of an endogenous gene. For such remarkable discovery, the authors were awarded the noble prize in physiology or medicine in 2006. Later, it had been found out that this silencing effect could be transmitted in the germ lines and thereby could be passed on to several generations of *C. elegans* (Fire et al., 1998; Grishok et al., 2000). This silencing phenomenon is also known as post-transcriptional gene silencing in eukaryotes (De Carvalho et al., 1992), co-suppression in plants (Napoli et al., 1990), and quelling in fungi (Romano and Macino, 1992).

**FIGURE 1:**
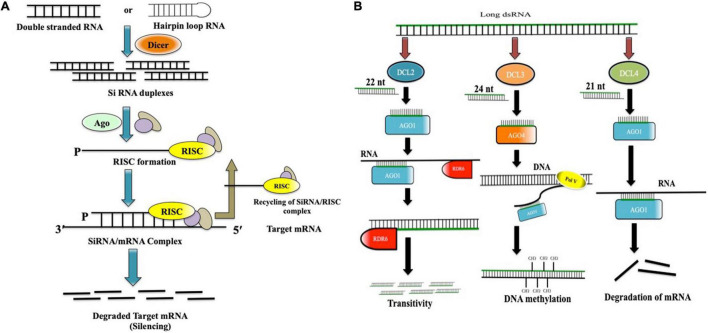
**(A)** In RNAi-mediated gene silencing, long dsRNA/hpRNA gets chopped off into short (21–23 nucleotides) interfering RNA duplexes (siRNAs). siRNA duplexes unwind and one of the strands (Guide strand) gets incorporated into the RISC complex, which finally degrades the targeted complementary mRNA. RISC complex gets recycled again; **(B)** Dicer-mediated processing of long dsRNA in *Arabidopsis thaliana*. DCL2 generates 22 nucleotide-long siRNAs, which interacts with AGO1 and recruits RDR6 to induce transitive silencing. DCL3 generates 24 nucleotide-long siRNAs, which interacts with AGO4 and induces DNA methylation by recruiting DNA PolV, whereas DCL4 generates 21 nucleotide-long siRNAs, which interacts with AGO1 and directs the cleavage of complementary mRNA.

There are mainly three types of non-coding RNAs, which are involved in the downregulation of gene expression: microRNAs (miRNAs), small interfering RNAs (siRNAs), and PIWI-interacting RNAs (piRNAs) (Hamilton and Baulcombe, 1999; Pasquinelli et al., 2000; Bartel, 2009; Aalto and Pasquinelli, 2012). Although all these three types have differences in their biogenesis, they all share a few similarities in their mode of action. There are mainly three steps in the RNAi-mediated gene-silencing process (Figure 1A). First, the long dsRNAs or the hairpin loop RNA (hpRNA) gets processed into 21–23 base-pair long nucleotide strand by Dicer (a ribonuclease enzyme type-III) (Ketting et al., 2001). *Arabidopsis thaliana* has four types of Dicer-like (DCL) paralogs. DCL1 is responsible for the synthesis of miRNAs, whereas DCL2, DCL3, and DCL4 synthesize 22, 24, and 21 nucleotide-long siRNA, respectively (Xie et al., 2004; Henderson et al., 2006). In the second step, these short duplex siRNAs unwind to form one guide strand, which is supposed to guide in targeting the complementary endogenous mRNA, and the other passenger strand, which eventually gets degraded. In final step, the guide strand gets associated with the argonaute proteins (AGOs) to form the RNA-induced silencing complex (RISC) and further guides the RISC complex to locate, bind, and degrade the complementary mRNAs in the cell (Ketting, 2011). Although it is noteworthy to mention that in Arabidopsis, processing of dsRNA varies greatly upon the interaction with each of the DCL paralogs. Generally, DCL4 generates 21 nucleotide-long siRNAs, which gets loaded on argonaute 1 (AGO1) and further guides in targeting complementary mRNAs in cytoplasm (PTGS) (Hamilton and Baulcombe, 1999; Mi et al., 2008). DCL2 generates 22 nucleotide-long siRNAs, which when loaded on AGO1, changes its conformation, and recruits RNA-dependent RNA polymerase 6(RDR6) to the 3′ end of the target, finally transcribing the target mRNA into another duplex siRNA (secondary siRNA). This process is known as transitive silencing (Dalmay et al., 2000). DCL3, on the other hand, creates 24 nucleotide-long siRNA, which when gets loaded with AGO4, recruits DNA methyl transferase, and triggers DNA methylation (Wassenegger et al., 1994; Chan et al., 2004; Figure 1B).

The basic mechanism and the key proteins involved in RNAi pathway are more or less evolutionarily conserved among prokaryotic and eukaryotic organisms (Shabalina and Koonin, 2008). It has been successfully applied to crops to confer resistance against a wide variety of biotic stresses such as insects, fungi, nematodes, and viruses. However, there are many drawbacks in using RNAi as a gene-silencing tool. For example, RNAi machinery is mostly active in the cytoplasm, and it is really difficult to target the long non-coding RNAs in the nucleus (Fatica and Bozzoni, 2014). A major concern over using RNAi is its potential risk of off-target silencing. It has been proved that siRNAs are capable of targeting and silencing non-target mRNAs which have a very few sequence similarities with the target mRNA (Carthew and Sontheimer, 2009). Another disadvantage is that sometimes, these off-target silencing results in a phenotype that dominates the required phenotype for which RNAi construct was designed on the first place (Franceschini et al., 2014).

#### RNAi for Improving Disease Resistance in Plants

Since its discovery, RNAi has been the primary choice for gene silencing across a wide array of biological fields, but inarguably, RNAi has been extensively used as a plant-protection platform over the years. Plants being a sessile organism have to withstand a wide variety of biotic and abiotic stresses that severely affects its growth and yield. Depending on the suitable environmental condition, various biotic stresses such as insects, fungi, and nematodes attack their host plants and cause severe loss in agricultural yield. Just like plants, insects have RNAi machinery primarily to mount defense against viruses (Matranga and Zamore, 2007). RNA interference in insects can be triggered by exogenous application of dsRNAs in plants by means of dsRNA injection or by feeding insects with dsRNA containing artificial diets (Baum et al., 2007) or by developing transgenic plants expressing dsRNAs targeting vital insect genes (Vélez and Fishilevich, 2018; Zotti et al., 2018; Di Lelio et al., 2022). Once this fact was established, researchers started concentrating their focus on improving the integrated pest management system by synthesizing dsRNAs targeting insects that cause severe damage to important crops. Here, we have discussed different approaches (including GMO/HIGS/SIGS) that involve “RNAi-as the central mechanism” to improve disease resistance in plants.

#### RNAi Against Insects

Brown planthopper *Nilaparvata lugens* is the one of the deadliest insect pests for rice. Li et al. (2011) were able to induce RNAi by targeting vacuolar ATP synthase subunit E of *Nilaparvata lugens* by means of dsRNA ingestion (Li et al., 2011). Cotton plant, expressing *CYP6AE14* dsRNA, was generated to acquire enhanced resistance to cotton bollworm *Helicoverpa armigera* (Mao et al., 2011). *In vitro* transcribed dsRNA was successfully applied in citrus and grapevine trees by injecting the trunks or drenching the roots, targeting the arginine kinase of two psyllids (Hunter et al., 2012). In a study Bolognesi et al. (2012) first characterized the mechanism of action of dsRNA targeted against the western corn rootworm larvae. The cotton aphid *Aphis gossypii* is a lethal pest to many agriculturally important crops that helps in virus transmission. They are also capable of developing resistance against a wide range of insecticides. This resistance against organophosphorus insecticides was reduced significantly by means of orally delivered dsRNA targeting carboxylesterase gene (Gong et al., 2014). Transplastomic potato plants (*Solanum tuberosum*), expressing dsRNAs, were developed that targeted the β-actin gene of the Colorado potato beetles (Zhang J. et al., 2015) and thereby showed significant resistance against this detrimental pest (Table 1).

**TABLE 1:** RNAi-targeted editing in plants against insects.

Insects	Target gene	Plant	Phenotype	References
*Helicoverpa armigera* (Cotton bollworm)	Ecdysone receptor	Tobacco	Reduction of growth followed by death	Zhu et al., 2012
*Diabrotica virgifera* (Western corn rootworm)	V-ATPaseA	Maize	Stunted growth	Baum et al., 2007
*Spodoptera exigua*	Ecdysone receptor	Tobacco	Death rate increased	Zhu et al., 2012
*Sitobion avenae* (Grain aphid)	Salivary proteins DSR32/DSR33	Wheat	Death rate increased	Wang et al., 2015
*Glossina morsitans morsitans*	Transferrin	Pea, clover, alfalfa	Mortality rate significantly low	Walshe et al., 2009
*Acyrthosiphon pisum*	Aquaporin	Pea, clover, alfalfa	Osmotic pressure increased	Shakesby et al., 2009
*Acyrthosiphon pisum*	*SHP*	Pea, clover, alfalfa	Reduced fertility	Will and Vilcinskas, 2015
*Acyrthosiphon pisum*	V-ATPase E	Pea, clover, alfalfa	dsRNA degradation in saliva	Christiaens and Smagghe, 2014
*Nilaparvata lugens* (brown plant hopper)	Trehalose PO4 synthase	Rice	Lethality	Chen et al., 2010
*Aphis gossypii* (Cotton aphid)	*AgOBP2*	Cotton	Failure of recognizing host	Rebijith et al., 2016
*Sitobion avenae* (Grain aphid)	Catalase gene *CAT*	Wheat	Survival rate reduced	Deng and Zhao, 2014
*Sitobion avenae* Grain aphid	Cytochrome c oxidase	Wheat	Increased mortality	Zhang et al., 2013
Greenbug *Schizaphis graminum*	Salivary protein C002	Wheat	Lethal	Zhang Y. et al., 2015
*Peregrinus maidis*	V-ATPase B&D	Corn	Reduced fertility	Yao et al., 2013
*Lygus lineolaris*	Apoptosis inhibitor	Cotton, alfalfa, beans	Digestion of dsRNA	Allen and Walker, 2012
*Phyllotreta striolata*	Arginine kinase	Cruciferae crops	Retarded development/increased mortality	Zhao et al., 2008

#### RNAi Against Fungi

In eukaryotic evolutionary hierarchy, the fungi are a major kingdom, which happens to have the RNAi machinery alongside the plants and animals. Although there are some exceptions, several lineages such as *Saccharomyces cerevisiae*, some close relative yeasts, and filamentous fungi such as *Cryptococcus gattii* and *Ustilago maydis* have lost their RNAi pathway during evolution (Drinnenberg et al., 2011; Nicolás et al., 2013). The reason for loosing RNAi was that *S. cerevisiae* and other filamentous fungi were infected with the “killer virus,” which kills neighboring cells that possess an active RNAi, while providing immunity to the host cell itself. Hence, the benefit of having RNAi was proved to be selective disadvantage, thus creating RNAi-deficient fungi (Drinnenberg et al., 2011). The RNAi mechanism in other fungi such as *Neurospora crassa* was first described as quelling by Romano and Macino (1992). Creating transgenic plants expressing dsRNA targeted against fungal genes soon proved to be a promising approach for plant protection (Koch and Kogel, 2014). In a study with barley, *Fusarium graminearum* infection was controlled by spraying 791 nucleotide-long dsRNA (CYP3-dsRNA) (Koch et al., 2016). Mycotoxins from fungus are considered to be very harmful for both plants and animals. The mycotoxin content of *F. graminearum* was significantly reduced by transforming it with inverted repeat transgenes (IRTs) containing mycotoxin-specific regulatory genes (McDonald et al., 2005; Table 2).

**TABLE 2:** RNAi-targeted editing in plants against fungi.

Fungi	Target gene	Plant	Phenotype	References
*Puccinia triticina*	*PtMAPK1*	Wheat	Significant reduction of fungal growth and disease suppression	Panwar et al., 2013
*Blumeria graminis* f.sp. *tritici*	*MLO*	Wheat	Inhibition of fungal growth	Riechen, 2007
*F. graminearum*	IRT containing mycotoxin regulatory sequences	Grain and legume crops	Significant reduction in mycotoxin production	McDonald et al., 2005
*Blumeria graminis*	*Avra10*	Barley and wheat	Inhibition of fungal growth	Nowara et al., 2010
*Phytophthora parasitica*	*PnPMA1*	Arabidopsis	Elucidation of a compatible interaction between Arabidopsis and *P. parasitica*	Wang et al., 2011
*Fusarium verticillioides*	*GUS*	Tobacco	Reduced expression of *GUS*	Tinoco et al., 2010
*Phytophthora parasitica* var. *nicotianae*	Glutathione S-transferase	Tobacco	Increase resistance of Nicotiana to infection	Hernández et al., 2009
*Puccinia striiformis* f. sp. *tritici*	*PSTha12J12*	Barley and wheat	Significant improvement in rust resistance	Yin et al., 2011
*Botrytis cinerea*	MAP Kinase *Bmp3*	Lettuce	Delay in conidial germination, reduction of necrotic lesions	Spada et al., 2021
*Botrytis cinerea*	*DCL1*, *DCL2*	Tomato, Strawberry, Grape, Lettuce, Onion, Rose	Significant inhibition in gray mold disease	Wang M. et al., 2016
*Botrytis cinerea*	*DND1*	Tomato and Potato	Reduced susceptibility to Botrytis	Sun et al., 2017
*Botrytis cinerea*	*BcTOR*	Arabidopsis, Potato, Tomato	Enhanced resistance against gray mold	Xiong et al., 2019

#### RNAi Against Nematodes

Since the discovery of RNAi in model nematode *C. elegans* Fire et al. (1998), an overwhelming amount of work has been done to further widen our knowledge regarding the nematode genomics. Out of several plant-parasitic nematodes (PPNs), root-knot nematode such as *Meloidogyne incognita* causes significant crop loss across the globe (Sasser et al., 1984). Among the several essential proteins required by root-knot nematodes, 16D10 was found out to be a very effective target gene for controlling the infection. This particular gene was identified and subsequently silenced using RNAi and thus suppressed nematode development (reproduction) by 74–81% (Huang et al., 2006). *Mi-Rpn7*gene is a potential target for controlling root-knot nematode *M. incognita*. Knocking down of *Mi-Rpn7* using RNAi resulted in 55.2–66.5% reduction in infectivity (Niu et al., 2012). Dutta et al. (2015) succeeded in making a transgenic tomato line targeting *M. incognita*-specific protease gene cathepsin L-cysteine proteinase (*Mi-cpl-1*). RNAi-mediated suppression of Mi-cpl-1 resulted in significant reduction in infectivity by the nematode (Dutta et al., 2015; Table 3).

**TABLE 3:** RNAi-targeted editing in plants against nematodes.

Nematodes	Target gene	Plant	Phenotype	References
*Meloidogyne incognita*	Splicing factor and integrase	Tobacco	>90% reduction in established nematodes	Yadav et al., 2006
*Heterodera glycines*	*Prp-17, Cpn-1*, mRNA splicing factor	Soybeans	Significant reduction in number of nematode eggs	Li et al., 2010
*Radopholus similis*	*Rs-cb-1*	Tobacco	Inhibition of development, reduced pathogenicity	Li et al., 2015
*Ditylenchus destructor*	*Unc-15*	Sweet potato	Reduced rate of infection area	Fan et al., 2015
*Meloidogyne enterolobii*	*MeTCTP*	Tomato	Attenuation in parasitism	Zhuo et al., 2017
*Pratylenchus vulnus*	*Pv010*	Walnut	Significant reduction in number of nematodes per root	Walawage et al., 2013
*Heterodera glycines*	Spliceosomal SR protein, ribosomal protein	Soybean	Significant reduction in number of female cysts	Klink et al., 2009
*Meloidogyne chitwoodi*	*Mc16D10L*	Potato	Significant reduction (∼68%) in number of egg masses	Dinh et al., 2014a
*Heterodera schachtii*	*3B05, 4G06, 8H07*, and *10A06*	Arabidopsis	23–64% reduction in number of mature nematode females	Sindhu et al., 2009
*Heterodera glycines*	Major sperm protein (MSP)	Soybeans	68% reduction in eggs per gram of root tissue	Steeves et al., 2006
*Meloidogyne javanica*	Putative transcription factor, *MjTis11*	Tobacco	Consistent silencing of *MjTis11*	Fairbairn et al., 2007
*Meloidogyne chitwoodi*	*Mc16D10L*	Arabidopsis	Significant reduction (∼60%) in number of egg masses	Dinh et al., 2014b

### CRISPR/Cas: The New Era of Genome Editing

**FIGURE 2:**
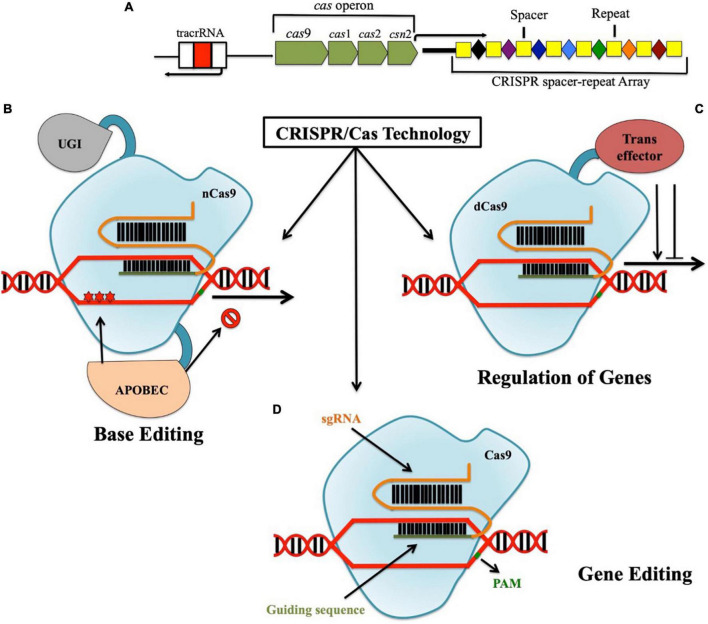
CRISPR locus in genome comprises of CRISPR spacer-repeat array, Cas operon, and tracrRNA. Apart from being able to specifically target nucleotides in the genome (gene editing), using impaired Cas9 enzymes such as dCas9 and nickase Cas9, gene regulation and targeted base editing without double-strand breaks can also be achieved, respectively. **(A)** CRISPR locus in genome; **(B)** CRISPR/Cas9-mediated gene editing; **(C)** CRISPR/dCas9-mediated gene regulation; **(D)** CRISPR/nCas9-mediated base editing.

Clustered regularly interspaced short palindromic repeats was first discovered as some sort of “unusual structure” by Japanese researchers in 1987 while working with the *iap* gene in *Escherichia coli* (Ishino et al., 1987). Later in 1993, it was first characterized by Francisco Mojica from the University of Alicante, Spain (Mojica and Rodriguez-Valera, 2016). It was detected in various bacteria and archaea and are thought to be involved in the regulation of their genes (Mojica et al., 2002). More than a decade later, it was described as a bacterial adaptive defense system that contains a CRISPR loci comprising of CRISPR, DNA-targeting spacers, and Cas operon (Bolotin et al., 2005; Barrangou et al., 2007; Figure 2A). There are three distinct stages through which CRSIPR/Cas mediated adaptive immunity works, namely, adaptation, maturation, and interference. In the “adaptation stage,” new spacers from the invading organisms such as virus get directly incorporated into the CRISPR array (Barrangou et al., 2007), which finally transcribes the precursor CRISPR RNA (pre-crRNA). With the aid of trans-activating CRISPR RNA (tracrRNA) coupled with CRISPR-associated csn1 protein and RNaseIII, these pre-crRNA becomes mature crRNA (maturation stage) carrying the individual spacer flanked by repetitive sequence. Finally in the “Interference stage,” these mature crRNAs along with Cas proteins form ribonucleoprotein (RNP) complexes that target and cleave foreign nucleic acid sequences, which are complimentary to the crRNA-encoded sequence (Deltcheva et al., 2011; Jinek et al., 2012; Zhang et al., 2012; Doudna and Charpentier, 2014). Since the discovery that CRISPR/Cas coupled with designed guide RNA can be used to specifically target and cleave any stretch of DNA sequence, it revolutionized the modern genetic engineering field (Jinek et al., 2012; Burmistrz et al., 2020). In fact, CRISPR-edited Sicilian Rouge tomatoes with high concentration of γ-aminobutyric acid (GABA) were the first genome-edited crop (approval in December, 2020) that entered the market of Japan successfully by Sanatech, a start-up from University of Tsukuba in September, 2021 (Waltz, 2022). Apart from conventional gene editing, using impaired Cas9 enzymes such as nuclease-dead Cas9 (dCas9; non-functional endonuclease activity) (Moradpour and Abdulah, 2020) and Cas9 nickase (nCas9; mutated nuclease domain of Cas9 for facilitating single-strand cut) (Adli, 2018), this powerful tool is capable of achieving gene regulation and targeted base editing, respectively (Chaudhuri et al., 2022; Figures 2B–D).

#### CRISPR/Cas System in the Generation of Pest-Resistant Crops

Invasion of plants by bacteria, fungi, nematodes, and all other sort of pests poses immense menace to agriculture. Application of agrochemicals and pesticides is the conventional source of resistance used by the farmers. But these agrochemicals gradually pass through the food chain and finally end up harming human health and cattle at large. Pathogens on the other hand are evolving at a steady rate to acquire immunity against these agrochemicals, which makes the production and use of agrochemicals a big challenge to society. One means to bypass this hurdle is the generation of pathogen-resistant plants through recombinant DNA technology where resistance genes from wild-type species can be introduced through standardized plant-breeding technologies (Wang F. et al., 2016). But sometimes alongside the desirable trait, unwanted traits also enter in the host genome. CRISPR has an excellent track record of genetic modification and has been successful in overcoming potential biotic stress challenges from fungi, bacteria, viruses, etc. (Pillay, 2020). A promising work by CRISPR/Cas9 on rice was the knockout of the gene-encoding mitogen-activated protein kinase-5 (*OsMPK5*), which enhanced plant disease resistance (Hillary and Ceasar, 2019). Researchers have found that conferring resistance to plants from RNA viruses is hard, because the spCas9 (nuclease from *Streptococcus pyogenes*) recognizes dsDNA solely. But with due perseverance, two enzymes were discovered, namely, FnCas9 (nuclease from *Francisella novicida*) and LwaCas13a (nuclease from *Leptotrichia wadei*), which can or could recognize RNA. As opposed to RNAi which confers GMO status, CRISPR/Cas9 has proven to be capable of implementing the modifications required to confer resistance against biotic stresses (in/del) at the nucleotide level. Following the laws of segregation, the CRISPR cassette gets discarded, finally generating plants without the GMO tag (Waltz, 2022).

#### CRISPR/Cas Against Viruses

Geminiviruses, the damaging family of DNA viruses, have always been an immense threat to plants. Recently, CRISPR/Cas technology has been successfully applied to plant system for achieving resistance against geminiviruses by three eminent scientific groups. Rep, coat CP proteins, and the conserved non-coding intergenic region (IR) are the essential components regulating the virulence of geminivirus. ORFs encoding these three components were targeted by Zahir Ali and his group using engineered sgRNAs, resulting in reduced viral load in the case of tomato yellow leaf curl virus (TYLCV) (Ali et al., 2015). A number of two other research groups also targeted various sequences of the geminivirus genome, which collectively reduced the virulence of the plant pathogen (Baltes et al., 2015; Ji et al., 2015). Zahir Ali also worked on three different viruses: TYLCV, beet curly top virus (BCTV), and Merremia mosaic virus (MeMV) simultaneously with engineered sgRNA using the same protocol mentioned above. The results suggest that all sgRNAs exhibit interference activity resulting in delayed or reduced viral DNA accumulation in the Nicotiana plant. Sanger sequencing also confirmed that TYLCV-specific IR-sgRNA containing the TAATATTAC sequence could target both TYLCV and MeMV genome, proving that a single sgRNA can simultaneously target multiple viruses (Ali et al., 2015).

#### CRISPR/Cas Against Fungi and Bacteria

Another potential destroyer of crop yield is fungal pathogens. Deadly plant fungal diseases such as smut, rust, rot, and mildew cause severe loss to agriculture every year. Over and above, the fatal mycotoxins from mycotoxigenic fungi, which accumulate as secondary metabolites in the tissues of the host plant, pose fatal health threats to human and animals from contaminated food and fodder. CRISPR/Cas9 approach is being applied nowadays to target the editing of potential candidate genes, which enhance plant resistance against fungal pathogens (Borrelli et al., 2018). Detailed survey reveals multiple bacterial plant pathogens that cause severe crop diseases which eventually result in heavy agricultural damage (Schloss and Handelsman, 2004). Since bacterial diseases are generally asymptomatic, so their control poses to be a humongous challenge for the agricultural scientists. The most common procedures adopted against bacterial diseases are biopesticides, genetic methods, etc. (Kerr, 2016). There are classifications among phytopathogenic bacteria. Some are crop-specific, e.g., *Clavibacter michiganensis* causing tomato ring rot disease, polyphagous-specific, e.g., *Ralstonia solanacearum* causing disease in dicots and monocots both together, and the last one being “kingdom crosser,” e.g., *Dickeya dadantii*, falling in the genre entomo-phytopathogen, attacking both plants and animals (Borrelli et al., 2018). Table 4 gives a detailed idea of the CRISPR/Cas9 tool used to combat DNA/RNA viruses, fungal and bacterial diseases for plant protection.

**TABLE 4:** CRISPR/Cas-targeted editing in plants against viruses, fungi, and bacteria.

Type	Pathogen	Target plant	Target gene and its function	References
Virus	Rice Tungro Spherical Virus (RSTV)	*Oryza sativa* *L. japonica*	Eukaryotic translation initiation factor 4G (elF4G). This is the host factor for the translation of RNA virus.	Macovei et al., 2018
Virus	Turnip Mosaic Virus (TuMV)	*Arabidopsis thaliana*	Eukaryotic translation initiation factor 4E (elF(iso)4E. This is the host factor for the translation of RNA virus.	Pyott et al., 2016
Virus	Cucumber Vein Yellowing Virus (CVYV), Zucchini Yellow Mosaic Virus (ZYMV), Papaya Ring Spot Mosaic Virus-W (PRSV-W).	*Cucumis sativus*	Eukaryotic translation initiation factor 4E (elF4E). This is the host factor for the translation of RNA virus.	Chandrasekaran et al., 2016
Virus	Bean Yellow Dwarf Virus (BeYDV)	*Arabidopsis thaliana* and *Nicotiana benthamiana*	Coat protein (CP), Replication Association protein (Rep), Intergeneric Region (IR). Carry our Rolling Cycle Amplification (RCA) mechanism.	Ji et al., 2015
Virus	Beet Severe Curly Top Virus (BSCTV)	*Nicotiana benthamiana*	Long Intergenic Region (LIR), Replication Association protein (Rep)/RepA. Carry our Rolling Cycle Amplification (RCA) mechanism.	Baltes et al., 2015
Virus	Tomato Yellow Leaf Curl Virus (TYLCV), Beet Curly Top Virus (BCTV), Merremia Mosaic Virus (MeMV)	*Nicotiana benthamiana*	Coat protein (CP), Replication Association protein (Rep), Intergeneric Region (IR). Carry our Rolling Cycle Amplification (RCA) mechanism.	Ali et al., 2015
Virus	Tomato Yellow Leaf Curl Virus (TYLCV), Merremia Mosaic Virus (MeMV), Cotton Leaf Curl Kokhran Virus (CLCuKoV)	*Nicotiana benthamiana*	Coat protein (CP), Replication Association protein (Rep), Intergeneric Region (IR). Carry our Rolling Cycle Amplification (RCA) mechanism.	Ali et al., 2015
Virus	Turnip Mosaic Virus (TuMV)	*Nicotiana benthamiana*	Coat protein (CP), Green Fluorescent protein 1, Green Fluorescent Protein 2, Helper Component proteinase silencing suppressor (HC-Pro)	Aman et al., 2018
Virus	Cucumber Mosaic Virus (CMV), Tobacco Mosaic Virus (TMV).	*Arabidopsis thaliana* and *Nicotiana benthamiana*	Open Reading Frame 1,2,3 (ORF 1,2,3), Coat protein (CP) and 3′ Untranslated Terminal Repeat (3′-UTR)	Zhang et al., 2018
Fungi	Rice Blast Disease (*Magnaporthe oryzae*)	*Oryza sativa L. japonica*	Ethylene Responsive Factor (ERF922). Several stress responses implicate transcription factor	Wang F. et al., 2016
Fungi	Rice Blast Disease (*Magnaporthe oryzae*)	*Oryza sativa L. japonica*	Exocyst Component Complex (SEC3A). This acts as the subunit of the exocyst complex in rice.	Ma et al., 2018
Fungi	Powdery mildew (*Blumeria graminis* f.sp. *tritici*)	*Triticum aestivum*	Mildew-Resistant Locus-A1(MLO-A1). This susceptible gene is associated with disease formation.	Wang et al., 2014
Fungi	Black Pod Disease (*Phytophthora tropicalis*)	*Theobroma cacao*	Non-expressor of pathogenesis-related3 (NPR3). Regulates the immune system.	Fister et al., 2018
Fungi	Powdery Mildew (*Oidium neolycopersici*)	*Solanum lycopersicon*	Mildew-Resistant Locus1 (MLO1). Responsible for the vulnerability toward this disease.	Nekrasov et al., 2017
Fungi	Powdery Mildew (*Erysiphe necator*)	*Vitis vinifera*	Mildew-Resistant Locus-7 (MLO-7). This susceptible gene is associated with disease formation.	Malnoy et al., 2016
Fungi	Gray mold (*Botrytis cinerea*)	*Vitis vinifera*	WRKY52. This transcription factor is associated with biotic stress response.	Wang et al., 2018
Bacteria	Bacterial Blight (*Xanthomonas oryzae* pv. *oryzae*)	*Oryza sativa*	SWEET13. This is the sucrose transporter gene.	Li et al., 2012; Zhou et al., 2015
Bacteria	Fire blight (*Erwinia amylovora*)	*Malus domestica*	DspE-interacting proteins of Malus (DIPM-1, DIPM-2, DIPM-4) involved in fire blight disease as a susceptibility factor	Malnoy et al., 2016
Bacteria	Citrus canker (*Xanthomonas citri*)	*Citrus paradisi*	Lateral Organ Boundaries (LOB1), gene promotes pathogen growth, pustule formation.	Jia et al., 2017
Bacteria	Citrus canker (*Xanthomonas citri*)	*Citrus sinensis Osbeck*	Lateral Organ Boundaries (LOB1), gene promotes pathogen growth, pustule formation	Peng et al., 2017

CRISPR/Cas9 technology for crop protection needs to be thoroughly explored by the researchers globally. It becomes an extremely handy tool for crop protection if it is already known what kind of modifications required in specific genes to confer disease resistance. Resistance to a certain disease can be achieved by CRISPR-mediated knockout of the target DNA and activation of the host cell’s DNA repair process [using non-homologous end joining (NHEJ)] to bring out the desired inactivating mutations. Thus, CRISPR can be proven useful in inactivating a single target gene or plethora of gene families using single gRNA that can target several sites or by multiplex method where multiple gRNAs are introduced simultaneously. There is another contrary case as well, where generation of resistance is caused by the involvement of particular allelic variants. Here, homologous directed repair (HDR)-based cell repair comes into action along with CRISPR-DNA cleavage. It has been inferred that the application of HDR has a promising potential alongside the NHEJ in insertion of mutations using CRISPR/Cas9 tool. Yet, multiple aspects need to be considered and worked upon if CRISPR/Cas9 technology is to be applied on a large scale in plant-pathogen defense scenario.

## Relevance of RNAi in Today’s World: In Terms of Exogenous Application of RNAi-Inducing dsRNAs

A tried and tested crop protection platform, the RNAi strategy, has been ruling the domain of plant biotic stress resistance for more than two decades. Initially, this technology was used to generate GM or transgenic plants (Table 5; Mat Jalaluddin et al., 2019) that expressed dsRNAs, overall which bestowed the plants with a natural defense mechanism having the capacity to resist invasions of viruses and other hostile organisms (Taning et al., 2020). It has been quite a while that the production of GMOs/transgenics has led to a lot of hue and cry all over the world. In addition, as goes the age old saying that “Necessity is the mother of invention,” the huge uproar of the public and the anti-GMO scientific community led to the discovery of alternative approaches that skip transgenics and directly apply RNA molecules (dsRNA-containing end-use products, dsRNA-EPs) exogenously to trigger the required RNAi response. Various methods of exogenous application such as spraying, root/seed soaking, mechanical inoculation, trunk injection, and petiole absorption are adopted by the scientists as alternative to the controversial GM strategy of knocking down target genes to control plant damage by biotic stress (Dalakouras et al., 2020). Exogenously applied dsRNA on plants has the capacity to initiate RNAi-mediated gene silencing of the invading pest or the pathogen genes. The researches by various scientific groups (Koch et al., 2016; Konakalla et al., 2016; Kaldis et al., 2018) have demonstrated that the dsRNAs are actively absorbed by the plant cells and are processed into siRNAs which consequently extend the time of prevention of some plant diseases. The efficacy of the RNAi-based strategies for pest control is dependent on multiple factors such as the mechanisms of host–pathogen interactions, structural characteristics, RNAi machinery, and so on. Some shortcomings of exogenous RNAi application have been noticed in case of some pathogenic fungi, where dsRNA is not absorbed by the specific pathogen and secondary siRNA amplification is absent (Song et al., 2018; Kettles et al., 2019). The effectiveness of the exogenously applied RNAi also depends on the mode of delivery of the dsRNA biomolecules, the most common modes being spray method of mechanical inoculation. This mode of delivery varies as per the type of plant selected and the targeted pest (Dalakouras et al., 2018). Exogenous application of naked dsRNAs, liposome-protected dsRNA, and artificial extracellular vesicles (EV) that might be complexed with protein carrier or nanoparticles has brought out significant downregulation of transgenes and various plant endogenes according to many reports (Jiang et al., 2014; Numata et al., 2014; Lau et al., 2015; Dalakouras et al., 2016; Huang et al., 2019).

**TABLE 5:** List of RNAi crops commercialized successfully (till 2021).

Crop	Year of approval	Targeted Gene	Trait
Tobacco [Vector Tobacco (USA) Ltd Vector 21–41]	2006	QPTase enzyme	Resistance against plum pox virus, Reduced nicotine
Plum (USDA-ARS- Appalachian Fruit Research Station C5)	2009	*CP* gene of PPV	Resistance against Plum pox virus
Papaya (University of Florida X17- 2)	2011	*CP* gene of PRSV	Resistance against papaya ringspot virus
Soybean (Monsanto MON 87705)	2014	*FATB1-A*, *FAD2-1A*	High oleic soybean oil, Low saturated fatty acid
Alfalfa (Monsanto/Forage Genetics KK179)	2014	*CCOMT*	Reduction in lignin content
Apple (Okanagan specialty fruits NF872)	2014	*Apo5, Ppo2*, *Gpo3*, *pSR7*	Non-browning
Potato (J. R. Simplot E12, E24, F10, F37, J3, J55, J78, G11, H37, H50)	2014	*Asn1* and *Ppo5*	Reduction in black spot, low acrylamide potential
Potato (J. R. Simplot W8)	2014	*Rpi-vnt1*, *R1*, *PhL*, *Vinv*	Late blight resistance, low acrylamide potential, reduction in black spot, lowered reducing sugar
Maize (MON 87411, Monsanto)	2015	*DvSnf7*	Western corn rootworm resistance
Potato (J. R. Simplot V11)	2015	*PhL, R1, Ppo5, Asn1*	Lowered reducing sugar, reduction in black spot, potential blight resistance, late low acrylamide
Apple (Okanagan specialty fruits GD743 and GS784)	2016	*Apo5, Ppo2*, *Gpo3*, *pSR7*	Non-browning
Potato (J. R. Simplot X17 and Y9)	2016	*PhL, R, Asn 1, Ppo5, Rpi-vn1, Vinv*	Lowered reducing sugars, low acrylamide potential, late blight resistance, reduced black spot

In the present-day scenario, the use of nanocarriers becomes highly prevalent commercially for crop protection against pathogens. They are predominantly used as sprayable RNA-based biocontrol pesticides (Kunte et al., 2020). So, RNAi technology and nanotechnology have been merged effectively to produce maximum benefits in pest and pathogen control industries. A mention worthy progress from laboratory to field is the generation of Bioclay or “layered double hydroxide clay nanoparticles” used as a potential vehicle to deliver or apply dsRNA (Mitter et al., 2017; Fletcher et al., 2020). Recently, it has been reported that DNA nanostructures can act as efficient RNA carriers using Watson and Crick base pairing and can serve as an excellent tool to deliver siRNA in plant cells (Zhang et al., 2019). A lot of research is being carried out regarding RNA-based biocontrol compounds, and most likely, they will be applied in the field of agriculture especially in greenhouse purposes using the appropriate delivery methods.

## RNAi-Based Biopesticides: Commercialization, Regulatory Guidelines, and Acceptance by Public

Research and analysis of the RNAi-based biocontrol sector have unfolded its immense potential in the development of the agricultural sector, off course with a patenting trend. With the formulation, discovery, patenting, and commercialization of sprayable RNA-based biopesticides, the recent investments in the research and development (R&D) of RNA based co-formulants have increased drastically (Table 6; Mat Jalaluddin et al., 2019; Bramlett et al., 2020; Christiaens et al., 2020; Giudice et al., 2021). The flag bearer here is the global giant Monsanto whose brand “Biodirect” is already in the process of developing RNA-based biopesticides to control pests, followed by other multinationals such as Bayer, Syngenta, and so on (Taning et al., 2020). The interest shown by these multinational companies in the domain of RNA-based biopesticides has motivated a plethora of agritech start-up companies to develop and function by utilizing the existing technology to curve their own niche in this agribusiness sector. Start-ups such as Greenlight Biosciences, AgroRNA, and RNAgri have started a humongous production of dsRNA at an affordable price. Greenlight Biosciences have developed an innovative and fruitful cell-free bioprocessing technique, which generates RNA sequences in a completely scalable method that will guarantee the availability of dsRNA at affordable rates for field application without bearing the GMO tag. Another budding start-up company AgroSpheres has emphasized on the protection of dsRNA against degradation along with enhanced target delivery to achieve maximum crop protection. This has been achieved by developing bioparticles made up of miniscule spherical cells that do not possess any chromosome and can encapsulate dsRNA. Recent reports state that GreenLight and AgroSpheres have collaborated to further work on the above-mentioned bioparticles to create a protective shield around the dsRNA to prevent degradation from RNases and ultraviolet radiations. Inspired by these recent developments, another biotech start-up Nanosur has specialized in producing modified RNA (MdsRNA) products that can be translocated smoothly and fast through cell membranes, thus improving efficacy and minimizing degradation. Following the footsteps of Nanosur, another start-up TrilliumAg has started its own agriculture platform called Agrisome, which excels in production of self-assembled protein-based nanoparticles called modified RNA molecules (MV-RNA), possessing improved stability and efficient target delivery. These RNAi-based biopesticides have become a great hit among public since they spare the agricultural crops from the GMO tag (Shew et al., 2017). Utmost endeavor has been made by the researchers to make these biocontrol methods a perfect fit into the current integrated pest management system which have been strategized to protect crops from biotic stress and be least depended on conventional methods with adverse effects (Taning et al., 2020).

**TABLE 6:** List of patent applications related to RNAi-inducing exogenous application of dsRNAs.

Application number	Invention details	Assignee	Category	Field of application	Legal status
CA 2790211(2011) CN201180012795 (2011) EP20110753916 (2011) US 13/042,856 (2011) US 14/015,715 (2013) US 14/015,785 (2013) US 13/583,302 (2011) EP20170152830 (2011) PCT/US2011/027528 (2011)	Regarding topical application of mixture containing RNAs, organosilicone surfactant and cationic lipid applied onto surface of plants	Monsanto Technology LLC	Industry	Herbicide resistance	Granted in US (2015), CN (2015) and EP (2017)
CA 2848680 (2012) CN 201280054179 (2012) EP20120832415 (2012) US 13/612,941 (2012) PCT/US2012/054862 (2012)	Regarding a process and composition for modulating Acetyl- CoA carboxylase in weed species	Monsanto Technology LLC	Industry	Weed control	Granted in US (2016)
CA 2848685 (2012) CN 201280053985 (2012) EP20120831945 (2012) US 13/612,948 (2012) PCT/US2012/054883 (2012)	Regarding a process and composition for modulating glutamine synthetase in weed species	Monsanto Technology LLC	Industry	Weed control	Granted in EP (2017), US (2016)
PCT/US2016/014344 (2016)	Regarding insecticidal composition containing organosilicone surfactant/cationic lipid, dsRNA complementary to leptinotarsa genes	Monsanto Technology LLC	Industry	Pest control	Entry into the national phase
US 14/037,750 (2013) PCT/US2013/062293 (2013)	Regarding transfection of *A. tumefaciens* crude containing hpRNA targeting BCTV and related curtovirus genes in sugar beets and tomato	Secretary of agriculture, United States	Government	Virus resistance	Granted in US (2017)
PCT/EP2007/000287 (2007) CA 2627795 (2007) EP20070700223 (2007) EP20110157036 (2007) EP20110157045 (2007) US 12/087,537 (2007)	Regarding dsRNA spray carrying adjuvant and surfactant for insect control	Devgen NV	Industry	Insect control	Granted in EP (2012, 2017)
PCT/US2012/050687 (2012) CA 2842709 (2012) CN 201280039977 (2012) EP20120823842 (2012) US 13/585,947 (2012)	Regarding formulation of dsRNA, water, plant hormone Brassinosteroid applied to the leaf surface of plants	Syngenta Participations AG	Industry	Pest/pathogen control	Granted in US (2016), CN (2016) and EP (2016)
PCT/EP2004/013049 (2004) EP20040818796 (2004) US 10/579,503 (2004)	Regarding addition of naked, unpackaged dsRNA or siRNA to the feed of plant sap-sucking insect	CSIRO Bayer BioScience NV (Transfer of rights to Bayer in 2012)	Government, Industry	Pest control	Granted in EP (2011)
EP20140870784 (2014) PCT/AU2014/000255 (2014) CA 2934289 (2014) US 15/106,548 (2014)	Regarding a naked dsRNA loaded upon LDH clay nanosheets	The University of Queensland	University/Research Institute	Virus resistance	Application submitted
MY-154890-A (2007)	Regarding a process for protecting orchids from CYMM virus using bacterial crude containing dsRNA	University of Malaya	University/Research Institute	Virus resistance	National (MY) patent
US 11/107,370 (2005)	Regarding preparation of a solution comprising recombinant *A. tumefaciens* containing a nucleic acid sequence complementary to the sequence of a target virus	The Samuel Roberts Noble Foundation	University/Research Institute	Virus resistance	Granted in US (2009)
PCT/US2014/026301 (2014) US 14/776,583 (2014)	Regarding topical introduction of dsRNAs having a sequence identical to an EIN2 gene to plant surface	Monsanto Technology LLC	Industry	Delayed senescence in flowers	Application submitted
CA 2873828 (2013) CN 201380039346 (2013) EP20130794339 (2013) US 13/901,326 (2013) US 14/403,491 (2013) PCT/IL2013/050447 (2013)	Regarding introduction of exogenous RNA into seeds using a kit containing naked dsRNA and a priming solution	A.B. Seeds Ltd	Industry	RNA carrier	Application submitted
US 15/123,139 (2015) PCT/JP2015/057221 (2015) EP20150758536 (2015)	Regarding transformation of a plant using carrier peptide containing a complex of nucleic acid molecules and a penetrating polycationic sequence.	Riken	University/Research Institute	RNA carrier	Application submitted
PCT/IL2016/050877 (2016)	Regarding a method of delivery into a plant cell using a composition of polycationic polymers, anionic surfactants, cuticle penetrating agent and polynucleotides	Forrest Innovations Ltd	Industry	RNA carrier	Application submitted

After the discovery of a new product and before its approval for the commercial use, there lay a huge procedure to be followed and that is its potential risk assessment by various biosafety committees. Since dsRNA-based pest control method follows a novel mode of action, biosafety studies have been specially strategized to acquire accurate data regarding the target selectivity and all potential risks involving these novel dsRNAs (Arpaia et al., 2017). Risk assessment in this domain is mainly concerned with the adverse effects that might occur to the non-target organisms (NTOs) exposed, which might lead to the overall negative repercussions on the environment. The mode of action of these biopesticides should be thoroughly analyzed and their adverse effects on the NTOs should be inferred, so that those adverse chains of events can be minimized. When RNAi-inducing dsRNAs accumulate in optimum concentration in the system of these NTOs, it triggers the endogenous RNAi machineries within NTOs, resulting in unwanted gene knockdowns leading to health hazards. All possibilities of such events have to be ruled out through a series of experimental field trials to claim the RNA-based biocontrol compound to be safe for use. Some sequence-independent events might also occur inside the NTOs upon exposure to non-specific dsRNA, causing potential harm to them (Lundgren and Duan, 2013). Another major concern regarding biopesticides (dsRNA) is the formulations used to improve its retention power and delivery inside the target organism. NTOs overexposed with these newly formulated dsRNAs fail to degrade them with their intrinsic nucleases as the cumulative dose amount of formulated dsRNAs is far more magnified and various risk assessment committees have been formed to address several concerns regarding these RNA-based biopesticides. The Federal Insecticide, Fungicide, and Rodenticide Act (FIFRA) Science Advisory Panel took an active initiative to address the potential ecological risks and human health risks and the ways and methods to minimize both. The European Food and Safety Authority (EFSA) also has a commendable role in assessing the risk factors of RNAi-based GM plants. The crux of this discussion is if proper regulatory guidelines are demarcated, then RNA-based biopesticides can prove to be both cost-effective and time-saving to prepare as compared to synthetic pesticides.

In many countries in the world including a third world country such as India, major contribution to economy is made by the agricultural sector. A considerable loss in crop yield happens annually due to various environmental stresses and pest–pathogen attack. So, applications of these RNAi-based biopesticides are of utmost importance. The agriculture industry, as a whole, is a pretty complex structure with multiple layers of middlemen in every stratum. Starting from the R&D scientists who discover these novel biopesticides, till it reaches the hands of local farmers, the product has to pass through field trials, approval of proper biosafety committees for commercialization followed by manufacturing units of agritech start-up farms, distribution agencies, and through the rest of stakeholders. The utmost need of the hour is proper communication between every stratum of this complex sector, so that mass awareness can be created regarding the usage of these RNAi-based biopesticides. Earlier, the worldwide GM controversy has taught us all a valuable lesson that educating and enlightening the common man is generally least bothered about. So, the industry and the R&D sector are the most required bridge between both the farmers and the common consumers. They should come forward and clear out the air regarding all pros and cons of the newly launched biopesticides in the market. The ultimate aim should be to emphasize on the need of proper research and evidence-based product approval by authorized biosafety committees to introduce these novel tools to be implemented for a sustainable agricultural system.

## Conclusion

RNA interference, a simple natural mechanism of eukaryotes, has been vastly exploited and extrapolated in the field of agriculture. The products generated (by endogenous/exogenous application) and marketed vouch its immense potential in the domain of crop protection. This technique has already curved its own niche in integrated pest management and sustainable agriculture. On the other hand, relatively new breakthrough genome-editing technology CRISPR/Cas is on its way to prove its efficacy in making disease-resistant smart crops without a “GMO” tag. Novel strategies for the above two technologies are needed on an urgent basis globally, to guarantee food security for the present and future generations. RNAi crops generated through HIGS are gradually paving the way to alternative approaches such as exogenously applied dsRNAs. But before commercialization, all the products need to pass through the acid test of assessment and regulation by biosafety committees. Exogenously applied dsRNA or “Biopesticides” possess immense potential to address the societal concerns regarding chemical pesticides and reluctance toward GMOs. Its successful application in pest management makes RNAi still relevant today. The success of these biopesticides finally lies on mass awareness of public and effective communication to expedite their commercialization.

## Author Contributions

KH conceptualized and designed the manuscript. KH and AC wrote the manuscript. MZA, MM, and AD critically read and edited the manuscript. KH, AC, MZA, MM, and AD approved its submission. All the authors contributed to the article and approved the submitted version.

## Conflict of Interest

The authors declare that the research was conducted in the absence of any commercial or financial relationships that could be construed as a potential conflict of interest.

## Publisher’s Note

All claims expressed in this article are solely those of the authors and do not necessarily represent those of their affiliated organizations, or those of the publisher, the editors and the reviewers. Any product that may be evaluated in this article, or claim that may be made by its manufacturer, is not guaranteed or endorsed by the publisher.

## Abbreviations

AGO: argonaute
CRISPR: clustered regularly interspaced short palindromic repeats
DBD: DNA-binding domains
DCL: Dicer like
DSB: double-stranded breaks
dsRNA: double-stranded RNA
GenEd: gene edited
GM: genetically modified
HIGS: host-induced gene silencing
hpRNA: hairpin loop RNA
HR: homologous recombination
miRNA: microRNA
NHEJ: non-homologous end joining
PAM: proto-spacer adjacent motif
piRNA: PIWI-interacting RNA
PTGS: post-transcriptional gene silencing
RDR: RNA-dependent RNA polymerase
RISC: RNA-induced silencing complex
RNAi: RNA interference
RVD: repeat variable di-residue
SIGS: spray-induced gene silencing
siRNA: small interfering RNA
SSN: sequence-specific nucleases
tracrRNA: *trans*-activating CRISPR RNA.
